# Work and Family Pathways and Their Associations with Health for Young Women in Korea

**DOI:** 10.3390/ijerph192315704

**Published:** 2022-11-25

**Authors:** Yujin Kim, Hyeyoung Woo, Sinn Won Han

**Affiliations:** 1 Department of Sociology, Kangwon National University, Chuncheon 24341, Republic of Korea; 2 Department of Sociology, Portland State University, 1721 SW Broadway, Portland, OR 97201, USA; 3 Jeb E. Brooks School of Public Policy, Cornell University, Martha Van Rensselaer Hall, Ithaca, NY 14853, USA

**Keywords:** work and family, work–family pathways, transitions to adulthood, health, young women, Korea

## Abstract

The aim of this study is two-fold: to discern patterns in pathways of work and family transitions among young women (aged 24–39 years) whose decisions and behaviors toward labor force participation, marriage, and parenthood are considerably shaped by social constraints and gender norms; and to examine whether and to what extent work and family pathways are associated with later health. Using data from a longitudinal survey based on a large sample of adult women in Korea (*N* = 2418), we identified eight dominant pathways of employment, marriage, and parenthood among young women and found that educational attainment and family values are strong predictors of these work–family pathways. We also found that the timing and sequencing of work–family pathways appears to be associated with later health outcomes. In particular, unemployed women who are not married and do not have children seem to be vulnerable to health problems, compared to those with other pathways. We discuss the implications of our findings regarding the occurrence of work and/or family transitions, as well as their timing and sequencing for women’s health in later life.

## 1. Introduction

Over recent decades, women’s labor force participation has continued to increase in many industrialized countries, while marriage and fertility rates have steadily declined [1]. These changes in women’s employment and family formation behaviors have been largely driven by their increasing educational attainment. In fact, as many women, especially younger ones, are more likely to have a college level education than their older counterparts, labor force participation is commonly expected once they complete their education [2]. However, owing to varying degrees of cultural expectations, social norms, and support and resources at both individual and institutional levels around the roles in work and family domains [3], combining work–family roles for women can still be challenging.

Entering the workforce and forming a family through marriage and/or parenthood are critical life transitions that influence an individual’s health and well-being in various ways. Not surprisingly, ample research has examined health consequences of these transitions among individuals, and the overall patterns of how employment, marital status, and parenthood are associated with health are relatively well known [4,5,6,7,8,9,10]. However, there is a lack of information about through which and to what extent work and family pathways account for individuals’ health differently over time.

Individuals’ decisions to enter and/or exit the labor force are often motivated by changes in their family status and circumstances. Of course, decisions to marry or divorce are also directly or indirectly induced by finance (e.g., employment). Additionally, although non-marital births and childless married couples have been on the rise in recent decades, most children are born to married women, rather than unmarried women, and once married, people are more likely to have children, instead of staying childless. Moreover, while younger generations tend to have fewer children than previous generations, modern parenting has become more expensive and time-consuming [11]. Combined with the competitive nature of the work environment and the precariousness of the labor market in contemporary society, performing multiple roles as a worker and a parent can be very challenging. Given the interconnectedness of the decisions of work and family transitions and the role performances in the work and family domains, it is imperative to consider employment, marriage, and parenthood simultaneously, rather than separately, when estimating the health consequences of these important life transitions [4,9,12].

This study focused on young women in Korea. Women’s health is an important public health concern in many countries, including Korea. Often expressed as “gender paradox” (e.g., [13]), women tend to live longer compared to men; however, women are more likely to report lower levels of health than men do. Despite high levels of educational attainment among young women—higher than young men in some countries—young women’s health is still lower than that of young men, and the lower levels of health among young women imply that some aspects of social roles and cultural norms in workplaces and family relations imposed on women, even young ones, may still not be in favor of their health. We believe that for young women in Korea this may particularly be the case, given that the levels of gender equality and glass ceiling index in Korea are the lowest among OECD countries [14]. In fact, the labor force participation among women in their 30s and 40s remains low—lower than many developed countries—despite the much-increased educational attainment of women in recent decades in Korea [15]. Drawing on these contexts, young women in Korea will serve as a unique case to elucidate patterns of interdependent relationships of family roles (employment, marriage, and fertility), and to examine the associations of family pathways with health in later life, offering meaningful implications of work–family transitions for not only the health of women but also overall population health. 

The primary purpose of this study is two-fold. First, we attempted to discern patterns in the pathways of work and family transition among women. To do this, we employed latent class analysis (LCA) to identify the major pathways that young women take for work and family. Second, once we identify the pathways, we examine whether and how women experience their health outcomes associated with these pathways from a life course perspective. To do this, we used a series of regression models with data from a longitudinal survey of a large sample of adult women in South Korea (hereafter Korea). 

## 2. Background

### 2.1. Life Transitions and Pathways

Young adulthood in contemporary society is typically characterized by several critical life transitions, such as completion of education, entering the labor force, having their own place to live, getting married, and having children. These transitions often entail additional social roles and responsibilities, which may influence other transitions and affect one’s life in various directions. According to the life course perspective, life pathways reflect patterns in the timing and sequencing of life transitions and their inter-connected social roles over the life course [16,17], and social roles have different meanings and consequences depending on where they fit within life pathways [12]. 

In general, the cultural context provides normative expectations and guidelines for schooling, labor force participation, and how and when family roles should be fulfilled and combined with other social roles [17]. In the contemporary post-industrial context, women (as much as men) are expected to have higher levels of education and contribute to the household income by participating in the labor market. Concurrently, young women are expected to marry by certain ages, and once married, having children is encouraged. Moreover, mothers are often assumed to be the primary caregivers of their children. 

Importantly, the combinations of multiple roles and timing and sequencing of work–family transitions may be influenced by education levels, available resources, and attitudes among women [18]. Women with a college degree or higher level of education are more likely to stay in the labor force and develop a career. They are also more likely to get married at later ages and have children while married. However, women without college degrees are likely to be guided by more traditional family and gender-role attitudes, which may lead to earlier transitions to marriage and parenthood. Given relatively limited resources, they may not have the option of combining work–family responsibilities, facing the decision to choose one between work and family, especially when they have minor children at home [18].

### 2.2. Employment, Family, and Health of Young Women 

There is robust evidence that employment has beneficial effects on the health of unmarried women (e.g., [19,20]). In addition, employment not only brings economic rewards but also provides opportunities to improve self-esteem and confidence and to expand social networks, all of which improve health conditions [21]. However, it is unclear whether this association holds for married women in the same way as for unmarried women [22,23]. To make a married life work, those in the marriage are expected to be committed to each other by being supportive and creating time and space for each other. Additionally, unless housework is equally divided or outsourced, wives often take on more household chores than husbands, resulting in an increased workload. Increased levels of time commitment and workload are likely to be even greater when they have children. Putting these links together, while employment may improve individuals’ health in general, for women this salutary association between work and health is often diminished or canceled out by higher levels of time constraints and role conflicts from the demands of marriage and parenthood [9,10,24]. 

Regarding women’s transitions to marriage and parenthood, ample studies have revealed several ways that it may affect the health of women. Overall, marriage is beneficial to women’s health, but parenthood is probably not [4,5,6,7,8,9,10]. Marriage may positively affect health by promoting healthier lifestyles and active social engagement [25,26,27] (see also [28]). Married women may enjoy additional income from their spouses and experience smaller economies of scale. They are also expected to have social and emotional support from each other, including intimacy, care, and daily interaction [10], all of which can enhance health and well-being. 

Similar to marriage, becoming a parent is a primary way of achieving an adult marker and fulfilling an expected adult role in many societies, where social norms about having children exist, and children may be an important source of enriching social ties and parents’ self-concepts and purposes of life [8,10]. However, young children create intensive daily demands on parents’ time, physical energy, and emotional care, which can contribute to and interrupt mothers’ careers and economic hardship in families [8,10]. Reflecting the complicated “rewards and costs” of having children, previous studies have shown inconsistent findings regarding the association between parenthood and health (e.g., [8]). On the one hand, children may improve parents’ well-being through expanded social networks, enhanced relationships with family and relatives, and stronger motivations to live a better life [8,29]. On the other hand, having children dramatically changes one’s life, with elevated levels of time, financial, and emotional demands, and creating role conflicts for mothers, especially working mothers, who may experience lower levels of health than those without children.

### 2.3. Joint Effects of Work and Family Transitions on Women’s Health

Education is a strong predictor of work–family transition. For example, compared to non-college-educated women, those with college education or more tend to enter the labor force with a “good” job, and get married at later ages and stay in the marriage [30,31]. However, in examining work–family transitions and their associations with health, many studies using longitudinal data consider point-in-time measures of a single transition over a relatively short follow-up period, without considering potential multiple transitions for a longer period. Additionally, while decisions on marriage and having children are highly contingent on employment status and work trajectories, especially for young women, these work and family transitions have rarely been treated simultaneously in previous literature. 

It is not uncommon for working women to put a stop to their careers or change their work from a full-time to a part-time job when they have minor children, either temporarily or permanently, because work may not be compatible with raising children. However, college educated women with a “good” job may have more advantages in reducing/avoiding role conflicts when adding the role of mother to their existing worker roles, as the good jobs may offer more resources that they can utilize. Conversely, less educated women with a “bad” job may encounter higher levels of role conflict than those with a “good” job, and they may not be able to afford to continue to work while caring for children (see [32] for meanings of good jobs and bad jobs). As such, education, work, and family formation are inter-connected directly and indirectly, implying that the protective and risk factors of each transition for work and family for health are manifested in complicated ways [33,34,35] (see also [36]): (1) which transition(s) in one domain (either work or family) are better or worse when combined with transition(s) in the other domain, and (2) whether health outcomes differ by when and in what order the work and family transitions occur. 

As expected, various types of work–family pathways are differently linked to health [4]. For example, those who make these transitions at “normative” timings in encouraged sequences (e.g., get a job in one’s 20 s, get married in one’s 30 s, have children in 1–3 years after the marriage while remaining married, etc.) may have more benefits for health from the transitions. However, those who either did not experience all of these transitions or did experience them in non-normative ways, concerning the timing and sequencing, may not enjoy the health benefits from the transitions as much. Additionally, those who did not experience any of the role acquisition through employment or family formation may face no health benefits or detrimental health, especially in a “tight” society, such as Korea, where having a strong norm on work–family transitions and non-normative types of work–family pathways are less well accepted [37]. Because social sanctions toward non-normative work–family pathways might still be strictly applied to women (even young ones) in Korea, women whose work–family transitions are not well timed and sequenced and thus follow “non-normative” pathways or those without any work–family transitions may experience lower levels of health, compared to women who sequentially transition into work, marriage, and parenthood [4,9].

### 2.4. The Case of Korea

As noted earlier, young women in Korea are important subjects for studying work and family pathways and their associations with health for several reasons. First, despite markedly increased levels of educational attainment among women over the past decades, their labor force participation rates have changed little [38]. In addition, M-shaped employment patterns, which are likely driven by unfair treatment in workplaces for women as well as rigid social norms regarding women’s obligations within marriage, were still observed even among women in their 30s and 40s [38]. Consequently, although Korea is known as a familial society where normative expectations about family formation are strong, increasing proportions of women among younger generations tend to delay their marriage and childbearing or remain unmarried and childless [38].

Scholars in Korea have also examined the association between family transition and health. Similar to the literature in the US, marriage is positively associated with health [39,40,41,42] (see also [43]). Yet, the health effect of motherhood varies by the age of children [44,45]. However, most previous studies usually considered one family transition at a time and examined its effect on health separately. In addition, although family transitions mostly occur during young and middle adulthood, they usually included older people as well since numbers of the never-married or divorced tend to be too small when limiting the analysis to younger people [39]. As results, it remains unclear how the transitions influence the health of young women. In Korea, the discrepancies among high levels of education, low labor force participation rates, continuously declining marriage and fertility rates, and persistent gender-role norms may have resulted in women taking the work–family pathway in more interesting ways. As some of these trends have not been observed before in many developed countries, it is imperative to understand the newly emerged work–family pathways among young women in Korea. In attempting to understand work–family pathways and their long-term consequences in women’s lives, this study also pays attention to the precursors of the pathways and women’s health in later life.

## 3. Materials and Methods

### 3.1. Data

Data for this study were obtained from the Korean Longitudinal Survey of Women and Family (KLoWF). Based on the 2005 Population and Housing Census, the KLoWF collected information from all women aged 19–64 years who lived in 9068 sample households across the country starting in 2007 (*N* = 9997). As a panel survey, the first two waves were conducted in 2007 and 2008; however, from the third wave onwards, the survey was conducted every other year, with the seventh survey in 2018 being the most currently available. We used all seven waves of the survey to investigate women’s family formation pathways and their associations with the consequences. One important advantage of the KLoWF is that it offers a wide range of information on childhood experiences, education, employment, women’s family life (i.e., marriage, fertility, and family member relationships), attitudes toward family and gender roles, and health over time based on a large sample of adult women in Korea.

To investigate women’s work–family pathways, we restricted our analytic sample to those aged 24 to 39 years and did not have a child in an unmarried status in the first wave (excluding 5877 respondents) to minimize potential endogeneity (i.e., selection bias). We also excluded respondents who had missing information on self-rated health in the seventh wave (excluding 1702 respondents), generating 2418 respondents for the current analysis (352 unmarried and 2066 married in the first wave). Through the selection process, our analytic sample of young women was likely to be more educated and healthier than the general population of women of the same age on average. We discuss how this may affect our results later in this study.

### 3.2. Measures

#### 3.2.1. Work and Family Pathways

We relied on three variables from Waves 1 to 6 to explore the types of family pathways: employment status, marital status, and whether they lived with a minor child. For employment, we coded 1 if the respondents worked for pay (either full-time or part-time) in each wave and 0 otherwise. If the respondents were married in a given wave, they were coded 1 for that wave and 0 otherwise. To determine co-residence with minor children (less than 18 years of age), we used information on resident biological children drawn from the respondents’ household rosters and examined whether the respondents lived with at least one minor child. If they lived with at least one minor child within the same household, we coded them as 1 for that wave, and 0 otherwise. “Living with a minor child” is not the same as parental status; however, we believe this variable captures women’s role conflict between work and family more efficiently, likely influencing subsequent decisions on work and family transitions over the life course.

#### 3.2.2. Precursor Variables

To measure family resources, we used three variables from Wave 1: parental education, childhood economic status, and family structure while growing up. For parents’ education, we used a higher level of education for fathers and mothers. Parents’ education had three categories: middle school or below (coded as 1), high school (coded as 2), and college or above (coded as 3). Childhood economic status was measured using the question, “What was your family’s financial situation around the age of 15 years?” Based on the answers to this question, we divided the respondents into three groups: financially difficult (coded as 1), average (coded as 2), and affluent (coded as 3). Family structure was determined using the question, “Did you live with your parents around the age of 15 years?” If respondents answered that they lived with both parents, they were coded as 1, and 0 otherwise.

Value orientations measured two dimensions: marriage and gender role. Four statements were used for each dimension. For marriage-related values and attitudes, we used the degree of agreement with the following statements: “everyone must get married,” “marriage should be with someone from a similar family background,” “it is better to get married early,” and “it is good to have children early if you are married.” A four-point Likert scale was used to measure respondents’ attitudes (strongly agree to strongly disagree). We reversed this original coding if necessary, so that higher values represented more conservative attitudes toward marriage. Attitudes toward gender roles were measured based on the following four statements: “ideally, men should have a job and women should take care of the household,” “housewives also have to go to work so that marital relations are equal,” “if housewives with preschool children work, it will have a negative impact on their children’s education,” and “the house couples live in must be under joint-ownership.” Higher values indicate more conservative attitudes toward gender roles. The educational level of respondents was categorized into three categories: high school or below (coded as 1), two-year college degree (coded as 2), and four-year college or above (coded as 3).

#### 3.2.3. Health

Health was measured using respondents’ self-rated health in Wave 7. Self-rated health was assessed with one question: “In general, how is your health?” (1 = very good, 2 = good, 3 = fair, 4 = poor, and 5 = very poor). In addition to the health at Wave 7, we also controlled for respondents’ health condition at the baseline measured at Wave 1. For both health measures, we reversed the original coding so that higher values indicated a better health status. 

#### 3.2.4. Other Controls

Two variables were included as controls. First, we considered the health behavior variable, which was measured based on the question, “In the past week, have you been doing strenuous physical activity for more than 10 min or more than usual? If yes, please tell us how many days have passed in the past week.” Based on the responses to this question, regular exercise was recoded into a variable with four categories: no exercise (coded as 1), 1–2 days (coded as 2), 3–4 days (coded as 3), and more than 4 days (coded as 4). Age at Wave 1 was also considered.

### 3.3. Analytic Plan

We proceeded with our data analysis in four broad stages [46]. In the first stage, we constructed a basic latent class model for repeated longitudinal data from Waves 1 to 6. Next, we assigned participants to latent classes based on their posterior probabilities. Third, we investigate the relationship between latent classes and precursor factors by employing a series of multinomial logistic regression analyses. Finally, we estimated how class membership is associated with self-rated health measured in Wave 7 by running OLS regression models with demographic and socioeconomic conditions adjusted for.

First, we used LCA to identify the differentiated types of pathways for our analysis. Work–family pathways are not well captured in a quantitative analysis because of several possible combined transitions between employment, marriage, and parenthood that have occurred at different times [47]. However, using LCA, which allows us to cluster observations into subgroups based on their patterns of response across a set of observed indicators, we can identify distinctive groups based on work–family pathways, offering a good picture of the types of pathways young women have taken in Korea. To estimate latent classes, we used three different role statuses (i.e., marital status, co-residence with minor children, and employment) across time and entered all 18 variables (i.e., three variables measured at six time points) as indicators of a single latent variable. We conducted a multiple-group LCA based on marital status (married vs. unmarried) in the first wave, since family formation pathways may differ for married and unmarried women over time. We also conducted LCA separately for married and unmarried women, but goodness-of-fit (i.e., Bayesian information criterion, log-likelihood values, G-squared) was better for multiple-group LCA models than for marital status-specific LCA models (results not shown here but available upon request).

To determine the optimal number of latent classes, we considered both goodness-of-fit statistics [48] and theoretical assumptions [49]. After much consideration (see Appendix A Table A1 for the goodness-of-fit statistics), we decided to select four-latent-class models for both married and unmarried women for further analysis. Once a four-latent-class model was selected, we conducted a measurement invariance test to examine whether the indicator response probabilities varied across groups (married versus unmarried). Our analysis indicated that one or more item-response probabilities are different across the two groups, implying that latent classes may have different meanings across groups [50]. 

After identifying eight work–family pathways (four pathways for married and unmarried women), we employed a series of multinomial logistic regression models to examine the predictor factors of the pathways, including family resources (i.e., parents’ education, childhood economic status, and family structure while grouping up) and value orientations (i.e., marriage and gender role). In addition, we estimated OLS regression models to examine the association of the pathways with self-rated health, with various confounders (i.e., health, health behavior, and socioeconomic status variables) controlled for.

## 4. Results

Table 1 presents the proportion of women who experienced the family transitions at each wave. For unmarried women in Wave 1, the proportion of married women living with minor children increased as more waves were observed. In contrast, the proportion of employment declined from 75% in Wave 1 to 53% in wave six. Next, as more waves were observed, the proportion of married women in Wave 1 living with minor children declined, while the proportion of employed women increased. This reflects the M-shaped employment pattern of women in Korea. While unmarried women’s employment grows, most married women with minor children temporarily exit the labor force and then return to the labor force later when their children get older. These proportions represent group averages; thus, they do not reflect the actual pathways of any particular group of women or the various family formation pathways. Below, we describe the LCA results.

### 4.1. Work and Family Pathways: LCA

**Figure 1:**
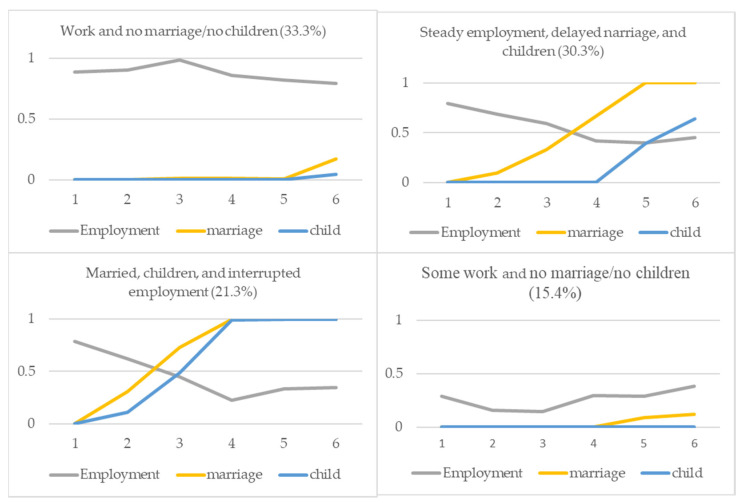
Unmarried women’s work and family pathways.

**Figure 2:**
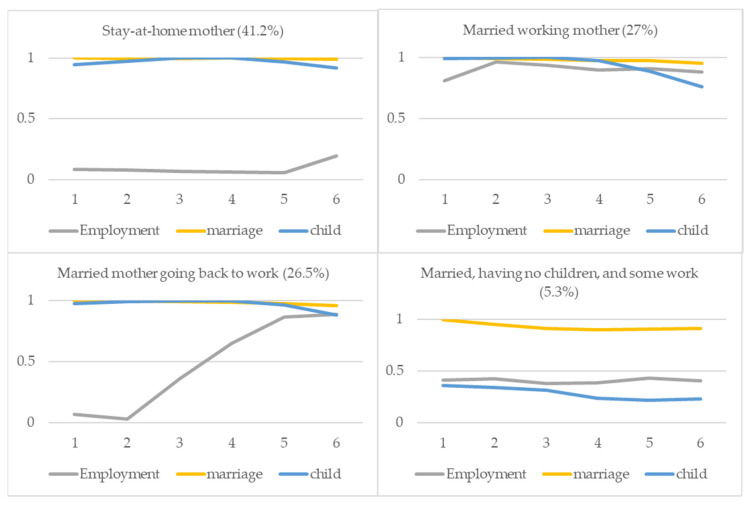
Married women’s work and family pathways.

Based on the results from the four-latent-class models, Figure 1 and Figure 2 present the item-response probabilities for the most likely latent class memberships among unmarried and married women in Wave 1. These figures show the expected role probabilities of the various inter-connected pathways of employment, marriage, and parenthood over time. First, for unmarried women in Wave 1, about three out of ten unmarried women belonged to the pathway of working without being married and having children, and this class was most likely to be observed among unmarried women. Since this group consists largely of single working women, we refer to this class as work and no marriage/no children. The next most frequently observed pathway was characterized as steady employment, delayed marriage, and children by working women who got married and then had children at later ages. Although the probability of being employed steadily declined after marriage and parenthood, women in this group stayed in the labor force at over 40% in all waves. Based on these two most frequently observed pathways, employment appears to be a key indicator of adulthood among young women in Korea. In contrast to the pathways driven by working women, we identified two classes that seem to be more family-oriented women. For example, approximately 21% of unmarried women were classified as married, children, and interrupted employment. This pathway was distinctive, with a high probability of marriage and rapid transition to parenthood after marriage. In addition, the probability of being employed declined sharply after marriage and parenthood, and it remained below 40%. Lastly, the least likely observed pathway was some work and no marriage/no children, featured by the low probabilities of marriage and parenthood in all waves. Many women in this group exhibited little transition toward family formation behaviors and low levels of labor force activities.

Next, we identify four classes of married women. As shown in Figure 2, the first class is for stay-at-home mothers: about four out of ten married women were on the pathway to be a stay-at-home mother, with high probabilities of being married and having children, but low probabilities of being employed. For women in this group, parenthood is coupled with only minimal involvement in employment, although their likelihood of being employed slightly increased in Wave 6. The second class was married working mothers, in which 27% of married women were in this class. Many women in this group played multiple roles simultaneously, with high probabilities of being married, having children, and being employed in all waves. For the third pathway among married women, a substantial percentage of married women, similar to those for the second pathway, the working married mothers, were on the pathway of being a married mother going back to work. In this class, married mothers returned to work after staying out of the labor force. Married mothers in this group were less likely to stay employed in earlier waves, but the probability of being employed continued to increase and reached almost 80% by Wave 6. Lastly, 5.3% of married women were defined by a high probability of marriage, but middle levels of employment and parenthood. We refer to this pathway as married, having no children, and some work. The characteristics of class memberships are described in Appendix A Table A2.

### 4.2. Precursors of Work and Family Pathways

To estimate the socioeconomic profiles of women and orientations toward marriage and parenthood across the eight classes identified earlier, we used multinomial logistic regression analyses. We conducted seven analyses for each class by comparing them with some work and no marriage/no children classes. As seen in Table 2, working women with no marriage/no children and those with delayed marriage and children were more likely to have a four-year college degree compared to women with some work and no marriage/no children. In addition, women with marriage, children, and interrupted employment were more likely to have a two-year college degree than women with some work and no marriage/no children. Family resources, including parental education level, economic status, and living arrangements, were not significantly associated with different family formation pathways. Unlike family resources, value orientation is significantly associated with different family pathways. Compared to women with some work and no marriage/no children, stay-at-home mothers and married working mothers were more likely to agree with the statements that “everyone must get married” and “it is good to have children early if you are married,” which suggests their traditional attitudes toward family formation.

### 4.3. Work and Family Pathways and Later Health

Table 3 presents the results for self-rated health regressed on class membership, controlling for sociodemographic, family background, and health behavior variables. As seen from Model 1, compared to some work and no marriage/no children classes, women from all other classes were more likely to have better self-rated health at Wave 7. We added sociodemographic and family background variables to Model 2, and the significant effects of family pathways remained constant. Age was significantly associated with lower levels of health, but education was not. Model 3, which is the full model, further includes the health and health behavior variables. Even after controlling for self-rated health in Wave 1 and current health behavior (i.e., regular exercise), family pathways still had significant effects on later health. Women who were inactive in family formation and employment were the most vulnerable group compared to women who experienced at least one adult role over the life course. However, the health differences across pathways with at least one adult role acquisition were not statistically significant.

## 5. Discussion

Considering the rapid changes in family formation and rigid gender-role norms in Korea, this study was designed to describe various pathways of work and family among young women and to examine whether and how diverse pathways are associated with women’s health in later life. We summarize our findings in three ways. First, by employing an LCA, we identified eight distinctive pathways of work and family transitions. Specifically, working women without family formation were most likely to be observed among unmarried women in Wave 1, while about four out of ten married women in Wave 1 were stay-at-home mothers. These two dominant pathways reflect that many working women tend to stay unmarried and childless and that many married women stay out of the labor force while they raise children. These pathways imply that most women in Korea face high levels of role conflict between work and family domains, where many workplaces are not family friendly and women carry much of domestic labor and care work for children and older parents at home. The labor market in Korea is highly competitive and precarious [51], and thus, it is hard to be an “ideal worker” while caring for and raising family and children. In addition, about 15% of unmarried women in Wave 1 followed the pathway of no family formation with limited employment during their late 20s to 40s. This group may be similar to the NEET (Not in Education, Employment, and Training) or idle (neither in social institutions—for example, school, work, marriage—nor in performing adult roles, such as a parent) observed in Western countries, reflecting the decline of some traditional pathways among women in Korea (i.e., entering labor market, then exiting it upon marriage and/or parenthood). 

Second, the pathways were affected by several factors, including individual-level resources, attitudes toward marriage, and gender roles. For example, educational attainment is a strong predictor of pathways: those with a college degree tend to stay active in the labor force, and when it comes to family behaviors, they are more likely to marry at later ages or remain unmarried. Those without a college degree are more likely to marry early, have children soon after, and tend to experience interrupts at employment. We also observe that those with strong family oriented values are more commonly found among mothers (regardless of whether they work for pay).

Lastly, the pathways are also associated with later health outcomes: particularly, women who are not married, do not have children, and are not active in the labor force report poorer health. Consistent with previous work [9], we did not find substantial differences among women who experienced any kind of adult role transition, including employment, marriage, or parenthood. The lack of significant differences between women in various work and family pathways does not imply that the timing or sequence of family formation is not associated with health. Instead, their effects on health are mitigated and conditioned by other events included in these pathways [9]. This may also imply that the health benefits of following a predetermined pathway—working for a short period until getting married and having children—might become smaller among young women in Korea, as more women navigate through various pathways. However, women with no adult role acquisition through family formation and work had the lowest levels of health, highlighting the importance of adult role acquisition in later health.

### Limitations

This study has several limitations. First, as indicated earlier, using a large sample of young women (aged 25–39 years), we selected those who did not have children outside of marriage and excluded some disadvantaged groups of women. Thus, we would like to call for caution when applying for young women in general, as our findings based on less disadvantaged women in this study are likely to serve as more conservative estimates, which underestimate existing health disparities across pathways. Second, given that we followed young women for their pathways over a 12-year span of time, we are aware that this period might not be sufficient to follow the entire work–family transition period during young adulthood. While indirectly, we attempted to cover the transitions as much as possible by looking at the respondents separately by marital status in Wave 1 (i.e., unmarried vs. married). However, we cannot completely rule out possible variations in the work–family pathway trajectories between and within these subgroups of our sample, which we were unable to capture. Third, as another data limitation, this study explored only women’s pathways of work and family formation since data on men were not available. Although the life course trajectories of men and women have become similar owing to reduced gender inequality in work and family domains, looking at the transitions to adulthood for both men and women and their health implications are important for understanding young adults’ lives. Finally, this study examined the health consequences of the pathways using self-reported health. The pathways of work and family transitions might be differently associated with other health outcomes, such as psychological and emotional well-being. We suggest that future studies contribute to this line of research by addressing these issues when feasible.

## 6. Conclusions

This study highlights that the timing and sequencing of work–family transitions have important health implications for young women in Korea. The timing and sequencing of life transitions are largely shaped by social institutions and cultural norms. Although the overall patterns in the timings and sequencing of work–family transitions have shifted and diversified in recent decades, our findings clearly suggest three messages. First, some types of pathways are more ideal than other types, at least for health. Given the higher levels of health observed among the women who made work and/or family transitions (e.g., those of the “work, delayed marriage, and children” in particular), compared to women who did not make any of the work–family transitions, young women seem to receive health benefits from both work and family. Second, when women perform roles in work and family domains, available resources from one domain may either reduce some role conflicts between the two domains or even facilitate role performance in the other domain. Lastly, and clearly not the least, our findings regarding the women who did not make all three transitions (i.e., entering the work force, getting married, and having children) imply that the current Korean society may expect young women to either participate in the labor force or form a family, if not both. However, the fact that many gender-inegalitarian and family-unfriendly work environments, combined with the increasing proportion of unmarried women, should be stressed as an important concern for women’s health and well-being in later life. For example, the Korean government should continue to increase gender diversity both in public and private sectors, especially at higher levels, as Korea is ranked at the bottom among the OECD countries, in terms of percentages of women in parliament and managers/directors. Also, the government should find ways to incentivize workplaces where work–family policies are well utilized. We urge that pursuing gender equity at workplaces may be the first step in promoting– the family transitions among young adults, which would improve the health of women (as well as men) in the long-term. 

## Figures and Tables

**Table 1:** Percentages of women in each work and family status at each wave.

Work and Family Status	Wave 1 (2007)	Wave 2 (2008)	Wave 3 (2010)	Wave 4 (2012)	Wave 5 (2014)	Wave 6 (2016)
Unmarried women *(N*)	310	272	270	258	268	284
Married	0	8.82	24.81	41.86	52.61	60.21
Living with minor children	0	2.26	10.32	21.29	33.23	42.26
Employed	74.52	66.45	62.26	50.32	50.65	53.23
Married women (*N*)	2066	1988	1961	1951	1960	1980
Married	100	99.4	98.93	98.46	97.96	96.72
Living with minor children	93.42	95.11	96.32	95.16	90.61	82.96
Employed	29.38	32.24	39.64	45.98	51.89	57.21

**Table 2:** Multinomial logistic regression of latent class membership regressed on precursor factors: B coefficient for each class compared with no family formation with limited employment.

	Work and No Marriage/No Children	Steady Employment, Delayed Marriage and Children	Married and Children, and Interrupted Employment	Stay-at-Home Mother	Married Working Mother	Married Mother Going Back to Work	Married, No Children, and Some Work
Education (ref. high school or below)							
Two-year college	0.525	0.831	1.299 *	0.366	0.286	−0.074	−0.055
	(0.59)	(0.58)	(0.65)	(0.48)	(0.58)	(0.51)	(0.65)
Four-year college or above	1.011 *	1.129 *	0.886	−0.417	0.473	−0.942 +	0.127
	(0.50)	(0.52)	(0.65)	(0.44)	(0.52)	(0.48)	(0.59)
Age (W1)	0.110 *	−0.071	0.020	0.383 ***	0.531 ***	0.377 ***	0.351 ***
	(0.05)	(0.06)	(0.08)	(0.05)	(0.07)	(0.05)	(0.06)
Self-rated health (W1)	0.016	0.219	0.199	0.348 +	0.526 *	0.257	0.124
	(0.22)	(0.22)	(0.26)	(0.20)	(0.26)	(0.21)	(0.23)
Structural resources							
Living standard at age 15 years	0.212	0.603	0.126	0.120	0.312	0.594 +	−0.029
	(0.34)	(0.38)	(0.43)	(0.29)	(0.33)	(0.31)	(0.41)
Parents’ education (ref. middle school or below)							
High school	0.160	−0.309	0.152	0.266	−0.084	−0.205	0.599
	(0.48)	(0.48)	(0.52)	(0.42)	(0.48)	(0.45)	(0.55)
College or above	−0.106	−1.003	−0.696	−0.851	−1.395 *	−2.050 ***	0.035
	(0.59)	(0.64)	(0.77)	(0.54)	(0.62)	(0.61)	(0.75)
Living arrangement at age 15 years	0.199	−0.191	0.693	0.700	0.970 +	0.372	1.409 +
	(0.62)	(0.67)	(0.89)	(0.49)	(0.58)	(0.53)	(0.75)
Value orientations							
Conservative marriage norm (W1, range 1~4)							
Everyone must get married	0.591 +	0.270	0.592 +	0.647 **	1.105 **	0.462 +	0.525
	(0.34)	(0.30)	(0.33)	(0.24)	(0.34)	(0.26)	(0.36)
Marriage should be with someone from a similar family background	−0.311	−0.338	0.043	0.144	−0.320	−0.148	−0.311
	(0.30)	(0.31)	(0.35)	(0.24)	(0.28)	(0.25)	(0.34)
It is better to get married early	0.021	0.020	0.189	0.367	−0.585	0.191	0.194
	(0.38)	(0.39)	(0.42)	(0.28)	(0.38)	(0.28)	(0.41)
It is good to have children early if you are married	0.160	0.178	−0.263	0.526 *	0.617 *	0.641 *	0.209
	(0.33)	(0.32)	(0.36)	(0.23)	(0.31)	(0.26)	(0.31)
Conservative gender-role attitude (W1, range 1~4)							
Ideally, men should have a job and women should take care of the household	−0.018	−0.067	0.214	0.112	0.095	−0.321	−0.002
	(0.31)	(0.30)	(0.31)	(0.23)	(0.27)	(0.26)	(0.34)
Housewives also have to go to work so that marital relations are equal	0.272	−0.580+	−0.851 *	0.084	−0.674 *	0.053	−0.456
	(0.29)	(0.32)	(0.39)	(0.24)	(0.29)	(0.24)	(0.35)
If housewives with preschool children work, it will have a negative impact on their children’s education	−0.267	−0.196	−0.992 **	0.365	−0.124	0.637 *	0.213
	(0.34)	(0.31)	(0.37)	(0.24)	(0.30)	(0.26)	(0.39)
The house couple live in must be under the joint-ownership.	−0.424	0.204	-0.043	0.216	0.239	0.098	0.153
	(0.28)	(0.30)	(0.32)	(0.21)	(0.23)	(0.23)	(0.29)
Constant	−3.794	1.756	0.032	−16.963 ***	−18.702 ***	−15.136 ***	−12.992 ***
	(2.40)	(2.90)	(3.15)	(2.53)	(2.99)	(2.45)	(3.08)
Observations	149	143	114	910	617	578	153

Standard errors in parentheses. *** *p* < 0.001, ** *p* < 0.01, * *p* < 0.05, ^+^ *p* < 0.1. ref.: reference. W1: wave 1.

**Table 3:** OLS regression analyses of latent class membership estimating self-rated health.

Variables	M1	M2	M3
Family formation pathways (ref. no family formation with limited employment)			
Work and no marriage/no children	0.368 **	0.378 **	0.359 **
	(0.12)	(0.12)	(0.12)
Steady employment, delayed marriage and children	0.520 ***	0.484 ***	0.451 ***
	(0.12)	(0.12)	(0.12)
Married, children, and interrupted employment	0.398 **	0.365 **	0.333 **
	(0.13)	(0.13)	(0.13)
Stay-at-home mother	0.342 ***	0.430 ***	0.410 ***
	(0.10)	(0.10)	(0.10)
Married working mother	0.319 **	0.435 ***	0.409 ***
	(0.10)	(0.11)	(0.10)
Married mother going back to work	0.327 **	0.425 ***	0.404 ***
	(0.10)	(0.10)	(0.10)
Married, no children, and some work	0.235 *	0.340 **	0.317 **
	(0.12)	(0.12)	(0.12)
Age (W1)		−0.017 ***	−0.014 ***
		(0.00)	(0.00)
Education (ref. high school or below)			
Two-year college		0.060	0.052
		(0.04)	(0.04)
Four-year college or above		0.060 +	0.057
		(0.04)	(0.04)
Living standard at age 15 years		0.000	−0.006
		(0.02)	(0.02)
Parents’ education (ref. middle school or below)			
High school		0.007	0.004
		(0.03)	(0.03)
College or above		0.022	0.024
		(0.05)	(0.05)
Living arrangement at age 15 years (ref. living with both parents)		−0.029	−0.051
		(0.05)	(0.05)
Self-rated health (W1)			0.126 ***
			(0.02)
Regular exercise (no exercise)			
1–2 days			0.014
			(0.05)
3–4 days			0.077 +
			(0.04)
≥ 5 days			−0.014
			(0.05)
Constant	3.375 ***	3.859 ***	3.293 ***
	(0.10)	(0.16)	(0.18)
Observations	2376	2376	2376
R-squared	0.009	0.021	0.043

Standard errors in parentheses. *** *p* < 0.001, ** *p* < 0.01, * *p* < 0.05, ^+^ *p* < 0.1. ref.: reference. W1: wave 1. M1: family pathway variable. M2: M1+ sociodemographic and family background variables. M3: M2+health and exercise variables.

## Data Availability

Not applicable.

## Appendix A

**Table A1:** Fit statistics for multiple-group latent class models.

Number of Classes	Log-Likelihood	G-Squared	AIC	BIC	Adjusted BIC	Entropy	df
1	−14960.93	11387.10	11459.10	11666.93	11552.55	1.00	524251
2	−12526.51	6518.27	6666.27	7093.48	6858.37	0.85	524213
3	−11646.20	4757.64	4981.64	5628.24	5272.39	0.89	524175
4	−11157.84	3780.91	4080.91	4946.89	4470.31	0.89	524137
5	−10866.71	3198.67	3574.67	4660.03	4062.71	0.91	524099
6	−10633.07	2731.38	3183.38	4488.11	3770.06	0.92	524061
7	−10459.51	2384.26	2912.26	4436.38	3597.6	0.93	524023
8	−10308.12	2081.48	2685.48	4428.98	3469.46	0.93	523985

**Table A2:** Descriptive statistics by each pathway.

	Work and No Marriage/No Children	Steady Employment, Delayed Marriage and Children	Marriage and Children, and Interrupted Employment	Some Work and No Marriage/No Children	Stay-at-Home Mother	Married Working Mother	Married Mother Going Back to Work	Married, No Children, and Some Work
Sample Size (n)	101	95	66	48	862	569	530	105
Age (W1)								
Mean	29.77	27.34	27.50	28.67	33.31	34.81	33.63	34.04
Range	24-39	24-39	24-34	24-37	24-39	25-39	24-39	24-39
Age (W7)								
Mean	40.72	38.33	38.42	39.67	44.31	45.81	44.62	45.04
Range	35-50	35-50	35-45	35-48	35-50	36-50	35-50	35-50
Education								
High school or below	25.74	17.89	16.67	43.75	48.49	49.21	54.53	55.24
Two year college	15.84	24.21	39.39	18.75	22.74	18.45	23.96	15.24
Four year college or above	58.42	57.89	43.94	37.50	28.77	32.34	21.51	29.52
Precursor Variables								
Structural resources								
Parents’ education								
Middle school or below	41.58	46.32	37.88	47.92	59.51	68.54	70.00	59.05
High school	37.62	40.00	50.00	31.25	29.81	23.37	23.96	30.48
College or above	20.79	13.68	12.12	20.83	10.67	8.08	6.04	10.48
Living Standard at age 15								
Low	20.79	14.74	15.15	31.25	27.49	29.17	27.92	32.38
Middle	53.47	62.11	63.64	52.08	55.22	54.31	52.83	47.62
High	25.74	23.16	21.21	16.67	17.29	16.52	19.25	20.00
Living arrangement at age 15								
Living with both parents	90.10	91.58	95.45	83.33	89.68	90.51	87.74	90.48
Others	9.9	8.42	4.55	16.66	10.33	9.49	12.27	9.52
Value orientations								
Conservative marriage norm (W1, range 1~4)								
Everyone must get married	2.32	2.38	2.47	2.13	2.51	2.57	2.49	2.35
Marriage should be with someone from a similar family background	2.82	2.81	2.92	2.90	3.03	2.96	2.98	2.92
It is better to get married early	2.07	2.00	2.12	1.94	2.30	2.25	2.36	2.19
It is good to have children early if you are married	2.48	2.52	2.44	2.42	2.91	2.83	2.97	2.55
Conservative gender role attitude (W1, range 1~4)								
Ideally, men should have a job and women should take care of the household	2.27	2.16	2.27	2.25	2.61	2.37	2.43	2.39
Housewives also have to go to work so that marital relations are equal	2.49	2.20	2.15	2.40	2.50	2.25	2.44	2.33
If housewives with preschool children work, it will have a negative impact on their children’s education	2.52	2.47	2.26	2.52	2.72	2.57	2.75	2.57
The house couple live in must be under the joint ownership.	1.73	1.83	1.68	1.88	2.07	2.11	2.06	2.09
Health and health behaviors								
Self-rated health (W1)								
Mean	4.11	4.32	4.29	3.98	4.02	4.04	4.02	4.02
Self-rated health (W7)								
Mean	3.74	3.89	3.77	3.38	3.72	3.69	3.70	3.61
Regular exercise per week (W7)								
no exercise	63.37	74.74	74.24	75.00	65.31	70.12	74.34	70.48
1~2 days	12.87	5.26	6.06	6.25	9.74	8.61	6.79	10.48
3~4 days	11.88	10.53	13.64	10.42	13.46	12.48	11.89	12.38
5+ days	11.88	9.47	6.06	8.33	11.48	8.79	6.98	6.67
